# Conceptualizing Processes of Agroecological Transformations: From Scaling to Transition to Transformation

**DOI:** 10.1007/978-3-030-61315-0_3

**Published:** 2020-09-22

**Authors:** Colin Ray Anderson, Janneke Bruil, M. Jahi Chappell, Csilla Kiss, Michel Patrick Pimbert

**Affiliations:** 6 Centre for Agroecology, Water and Resilience, Coventry University, Wolston, UK; 7 Cultivate! Collective, Bennekom, The Netherlands; 8 Centre for Agroecology, Water and Resilience, Coventry University, Coventry, UK

**Keywords:** Scaling up, Scaling out, Multi-level perspective, Sustainability transitions, Domains of transformation

## Abstract

In this chapter, we survey the recent literature that speaks directly to the issue of bringing agroecology to scale. We discuss the shift towards analytical frameworks that consider not only the farm level but rather whole food system transformations. We then introduce the multi-level perspective on sustainability transitions which we adopt for the purpose of this book. Moving beyond the technical analysis often found in research on sustainability ‘transitions’, our approach thus adopts agency-centric approach to food systems ‘transformation’. To do this, we introduce the notion of domains of transformation, which represent discrete areas where the conflict between agroecology and the dominant food regime manifests and where the potential for collective and transformation is transformation is most potent.

In recognition of agroecology’s multifunctional benefits and potential as a paradigm for the future of food, researchers, policy-makers and civil society organizations are converging around the theory and practice of scaling this system. They are looking at how food producers might be encouraged to adopt agroecology and, beyond that, at how agroecology can provide the framework for organizing and transforming entire food systems (IPES-Food 2016, 2018; Mier y Terán Giménez Cacho et al. 2018; IAASTD 2009).

Three dimensions to this process have been identified. In what is often called horizontal scaling out, “ever-greater numbers of families…practice agroecology over ever-larger territories”, engaging “more people in the processing, distribution, and consumption of agroecologically produced food” (Mier y Terán Giménez Cacho et al. 2018, p. 3). Others have argued for the importance of scaling up, in which changes that enable agroecology percolate through institutions, policies and law. A third dimension, deepening, involves seeking ever more synergies and improvements to the agroecological system itself. Yet all these dimensions present significant challenges, including asserting the political nature of agroecology in institutional spaces and policies.

One of the most commonly used frameworks for formulating transitions in agroecology is Stephen Gliessman’s (2005) five-level approach. The changes specified in these levels, it should be noted, do not generally unfold successively and neatly; there may be substantial ongoing overlap. At level 1, production is made more efficient by reducing the overall use of inputs (such as fertilizer, fuel or pesticides) across all types of farming systems, from conventional to organic. At level 2, external synthetic inputs are replaced with more sustainable ones, such as biofertilizers or organic pest management products, without fundamentally re-organizing the farming system. Mier y Terán Giménez Cacho et al. (2018) found that many successful transitions to agroecological systems on farms start with simple practices focusing on input substitution or incremental integration (e.g. new synergies between parts of a farming system) that produce benefits quickly (such as boosted yields or cost savings). The latter is important because it helps to motivate producers and may lay a path to more complex and extensive transitions. It is key to note, however, that top-down interventions by governments or aid agencies may view this kind of substitution as a final goal. However, on their own, Gliessman’s first two levels are unlikely to be transformative.

Level 3 is a step change: rather than minor tweaks to the existing farming system, it involves a redesign of the entire food and fibre production system based on ecological principles and natural processes. At level 3, multiple agroecological production practices (such as intercropping, compost, mixed farming) are reflexively introduced to foster the development of an intentional agroecological system. While such changes are envisaged as taking place at the farm level, they are deeply shaped by the wider context—the political, economic, cultural and social dynamics that help or hinder farmers’ capacity to act. Indeed, the complex integrations between components of the farm involved in agroecological redesign are often only possible when farmers are supported by relationships and structures beyond the farm such as territorial food markets, reciprocal labour arrangements with neighbours or wider diverse landscapes that foster insects and other pollinators. These often enable ecological, political and economic viability that would be difficult if not impossible to achieve as an individual farm. Thus, Mier y Terán Giménez Cacho et al. (2018) found that even in iconic case studies of agroecology transitions, level 3 integration is difficult, and currently relatively rare.

At level 4, connections between producers and consumers are strengthened to support the socio-ecological transformation of the food system. Here, the emphasis is on creating new markets for agroecological farm products and promoting solidarity between farms and their non-farming communities. An even deeper and wider transformation of policies, rules, institutions and culture occur at level 5, which focuses on social justice, democracy and other broad shifts.

While Gliessman’s framework has been picked up by the Food and Agriculture Organization of the United Nations (FAO) and many researchers and scientists, the dynamics at level 4 and 5 are mostly referred to in general terms: it is rare to see a concrete description of how transformations of this magnitude happen or the underlying power dynamics. Indeed, early academic work on agroecology was largely focused on the agronomic and ecological dimensions at the farm level (levels 1–3). This scope has shifted in the past five years, but there is still a need to better understand the wider social, economic and political processes that influence broader transformations (Fig. 3.1). Our work engages with the explosion of literature on agroecology and food system change to give nuance to these dynamics.

**Fig. 3.1:**
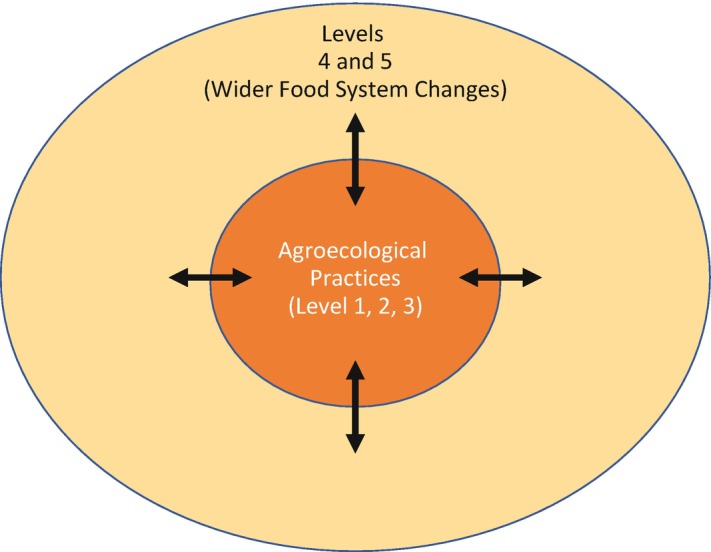
Steve Gliessman’s 5-level system has been used to conceptualize agroecology transition. The agronomic emphasis in early agroecology was historically focused on transition at the level of the farm, emphasizing understanding and enabling changes in farm practices (levels 1–3). In recent years, the reconceptualization of agroecology at broader scales and political agroecology as the basis for food-system change has centred analysis on levels 4 and 5. Our analysis in this book focuses on levels 4–5 to interrogate the wider social, political and economic dynamics that underlie the potential for food system transformation and its relationship with agroecological practices

We view food systems transformation as emergent, non-linear, context-specific, messy processes. The dynamics are not dissimilar to those of struggles towards, for instance, gender equality or environmental governance. Change in these arenas rolls out unevenly and uncertainly, with progress, repression, retrenchment, sudden breakthroughs and gradual changes. Progress is ever-evolving and may only be coherent in retrospect.

Thus, a large-scale transformation of food systems is actually *many transformations*, in which policy changes, struggles and networks that should be aligned are not always aligned (see, e.g., Holt Giménez and Shattuck 2011). “Hopeful monstrosities”—high potential but as-yet crude and inefficient performance—often emerge in these processes (Mokyr, J. 1990. The lever of riches: technological creativity and economic progress, New York: Oxford University Press. [Google Scholar], p. 291, cited in Schot and Geels 2008). Such imperfections are inevitable and may even be welcomed as creative experiments needing refinement in the midst of broader emergent transformational processes.

Agroecological transformations are thus not all or nothing. Often, progress can only be made through the kind of retrenchment, ‘blowback’ or inimical shifts. The process may involve a range of possible pathways, whose direction, speed and scale can be influenced but can never entirely be controlled by individual actors or actions such as state policies. So rather than adopting models such as linear levels of transition, or scaling up, scaling out or transition, we turn in the next section to the *multi-level perspective* *on sustainability* *transitions* (MLP) as one suitable, increasingly used framework for studying dialectical and emergent agroecological transformations.

The literature on the MLP is rooted in research on how ‘environmental innovations’ such as agroecology emerge and unfold. Within that field of study, sustainability transitions are considered “long-term, multi-dimensional and fundamental transformation processes through which established socio-technical systems shift to more sustainable modes of production and consumption” (Markard et al. 2012, p. 956). This tradition examines how environmental innovations have *replaced*, *transformed* or *reconfigured* existing systems.

Its focus is the dynamics, barriers and processes faced in the *transition* towards sustainability. But in this book we choose instead to embrace the idea of *transformation* in the sense of the shifts in power and governance that are central to a political agroecology. Such issues are rarely prominent in the literature on sustainability transitions. And sustainability transitions analysis in the agricultural sector has primarily focused on more technical aspects of the localization of food systems, organic agriculture or permaculture. In contrast, and as we have outlined elsewhere, choose to focus our conceptual framework on the critical issue of agency—that is, on the agency of people working in the fields, in grassroots organizations and social movements. We lift up the politics of the possible, where our analysis goes beyond critique to emphasize political possibility in spite of the grave structural barriers to the agroecology movement’s goals (Gibson-Graham 2006) and on frameworks, theories and ideas that strengthen agency and can mobilize action.

## Agroecological Transformations Within the Multi-level Perspective

In the literature on sustainability transitions, the multi-level perspective has been used to conceptualize dynamics and patterns in socio-technical transitions—such as in sustainable energy—as “non-linear processes that result from the interplay of developments at three analytical levels”. These levels are niches (the locus for ‘radical innovations’ and the development of alternatives), dominant regimes (the locus of established practices and associated rules that stabilize existing systems) and an exogenous landscape (events and long shifts outside these loci and timeframes, including macro-economic trends, political developments, wars and crises, the ongoing impact of “Deep cultural and societal values, and climate change”) (Geels 2011).

Originally, the MLP was developed as a way of understanding how technological innovation can lead to the consolidation of new commercial products in corresponding markets. But agroecology is more than a technological fix; it is an alternative paradigm and a political challenge to the status quo. In light of this, we avoid the technocratic framings of socio-technological regimes (Misra 2017), instead rooting our analysis in political dimensions.

Another body of literature, food regime theory (McMichael 2006), is helpful in the context of the MLP: because its perspective on transitions is explicitly political, it sheds light on global, historic antecedents and political ecological basis of today’s dominant food regime. These slowly unfolding landscape-level historical changes (elaborated below in the section on landscape level) set the historical foundations for today’s regime (see below section on regime level). Food regime theory also focuses on the semi-stable relationships of power between different actors (e.g. between civil society, government and the private sector); such relationships may also shift during the course of counter-movements (see below section on niche level).

By delving into the history of the current dominant regime, food regime theory has helped to unearth its colonial roots and ongoing dynamics, including racism, euro-centrism, patriarchy and capitalism, currently retrenched through corporate and neoliberal logics.

### Landscape Level

In the MLP, the *landscape level* represents macro-scale, often slow-moving contextual factors in society that do not necessarily directly determine change at regime or niche levels but rather “make some actions easier than others” (Geels and Schot 2007, p. 403). Examples of such factors include climate change, demographic change, shifting societal values and macro-economics. In a more specific example, massive shifts in power and sovereignty to corporate actors have been increasingly associated with international trade agreements which then embolden corporation’s intellectual property rights, making the spread of corporate-controlled commercial seeds easier and criminalizing many peasant seed networks.

Sudden crises, meanwhile, may often be manifestations of abrupt shifts in such slow-moving processes or by large-scale natural disasters. The worldwide anti-racism protests of 2020, for instance, are a manifestation of longstanding conflict between racial capitalism and the anti-racist counter-movement, ignited in response to the tragic murder of an African-American man, George Floyd, by the white policeman Derek Chauvin in May 2020. Periodic geo-political disasters, such as the food crisis of 2008 and the COVID-19 pandemic, are other recent examples of landscape-level crises that are generally beyond the direct influence of regime or niche actors but can play significant roles—for better or worse—in agroecology transformations. Such crises can be critical in sustainability transitions because they act as catalysts, creating pressure that can destabilize a regime and open up opportunities for alternatives to thrive (Mier y Terán Giménez Cacho et al. 2018). The opposite can, of course, occur: crises may give powerful actors in a regime an opportunity to reify their power and further entrench the status quo. For example, the ‘Mad Cow Crisis’ in the United Kingdom led to a further centralization and consolidation of power in the meat processing industry and food system, further undermining local food systems.

At the same time, an under-explored area of agroecology transformation is the role of ‘sparks’ other than crises that can ignite change. For example, John Kingdon’s classic 2011 work on political agenda-setting points out several precipitating factors (“focusing events”, such as disasters, crises or new discoveries) and opportunities for significant change (“windows”, when social and political actors are primed to act for change, such as elections or mass protests spurring society to action) (Kingdon 2011). Other precipitating factors can include dramatic changes in established indicators (e.g. the large increase in hunger and the use of foodbanks seen in recent years under austerity measures in the United States and the United Kingdom), influential new framings (e.g. discursive changes like ‘Black Lives Matter’ or ‘The 1%’—see Chap. 10.1007/978-3-030-61315-0_9 on the discourse domain) and the emergence and diffusion of a powerful symbol (the notion of extinction rebellion as a symbol to motivate action on climate change). Nor are crises enough to provoke agroecological transformation without preliminary political and organizational groundwork, as described by Eric Holt-Gimenez in the case of the deadly 1998 Hurricane Mitch. The best time to organize for transformation is before a crisis (or other ‘window’) manifests.

Food regime theory provides important analysis of broad shifts in the global political economic landscape over time that are particularly relevant for the landscape level. One commonality of all approaches to food regime theory is a central focus on how “forms of capital accumulation in agriculture constitute global power arrangements, as expressed through patterns of circulation of food” (McMichael 2009, p. 140). Food regime theory has remained centred, within the global historical context, on the contradictions and political struggles between different social groups in relation to food and agriculture. Those adopting the theory also generally accept that it focuses on the “rule-governed structure of production and consumption of food on a world scale” (Friedmann 1993, pp. 30–31). And they broadly agree on the existence of two food regimes, identified in early analyses.

The first, a ‘colonial-diasporic’ regime lasting from 1870 to the 1930s, was shaped by European colonialism and direct expropriation-dispossession from its colonies. The second ‘mercantile-industrial’ regime, running from the 1950s through the 1970s (and possibly into the present), was fundamentally shaped by the Cold War, the United States and its “informal empire of postcolonial states”, international developmentalist programmes and strategic uses of agricultural surpluses (McMichael 2009). As for now, Philip McMichael observes that a third, “possibly emergent”, regime has deepened the processes of the second regime. Alongside ongoing consolidation in food supply chains and food retail—this “emerging global food/fuel agricultural complex” is often termed the “corporate food regime” (Friedmann 2006).

Consistent with food regime theory, analysts who suggest that we are seeing a third regime identify it as a significant historical moment fundamentally shaped by tensions between “opposing geo-political principles” (McMichael 2009) in a corporatized “world agriculture”. In this regime, power has shifted away from the nation-state whereby a reconfiguration of power through neoliberal globalization and trade has given primacy to the global power of corporations in driving food regimes.

Grasping the dynamics of these landscape-level factors is important. However, it is in the interface between the niche and regime levels that the possibilities for agency are tangible and immediate.

### Regime Level

The *regime level* in the MLP represents “established practices and associated rules that stabilise existing systems” (Geels 2011, p. 26). Alignments and interdependencies of laws, processes, infrastructure and regulations across regimes tend to become locked in, building resistance to path-breaking innovations.

The thrust of today’s dominant food regime can be characterized by corporatization, a productivist (a reductionist focus on yield and profit) mentality, a reification of racist and patriarchal structural violence (e.g. the racialized and gendered patterns of poorly paid dangerous work around the globe) and an emphasis on monocultural, high-input, energy-intensive agriculture. This regime aims to standardize all aspects of food system to enable industrialization, decrease costs of production and increase profits. This push towards uniformity plays out not only in agronomics (monocultures of seeds, crops and livestock) but also in the erasure of diverse knowledge systems, markets and territorial agroecosystems that, as we will outline, are fundamental for agroecology and for sustainable food systems.

Powerful actors working on behalf of corporate and elite interests (e.g. lobbyists, politicians) also often attempt to resist or appropriate change in order to maintain the status quo. For example, where a government adopts a narrow technical understanding of agroecology into national frameworks, the system is at risk of being co-opted (Ajates Gonzalez et al. 2018). Structural power within a regime privileges particular actors at the expense of others along intersecting dimensions of oppression (e.g. racism, patriarchy, class).

However, this dominant regime has not been adopted universally over time and geographically; it manifests in different places in different ways, at different times, and is always met by resistance. In this context, transformations involve coalitions of actors involved in political, economic, cultural and social struggles and with competing interests, seeking to shape both the regime and emerging alternatives in particular places. A shift in power relations triggers the transformation—in particular, where disenfranchized actors and groups gain agency and power. In this book, we focus on agroecology as one specific form of resistance, which we further elaborate on in the next section as an emerging global niche.

### Niche Level: Agroecology and Food Sovereignty

Within (and against) this corporate food regime are social movements that are known as ‘niches’ within the context of the MLP. McMichael (2009) describes the dominant regime vis-à-vis the agroecological niche as “food from nowhere” (undifferentiated, appropriated and commercialized) and “food from somewhere” (grounded in place, space and culture)—that is, empowering local and traditional producers and food cultures.

In MLP parlance, niches represent the emergence of radical socio-technical alternatives to dominant principles and ways of working. This distinguishes them from ‘market niches’—specialized products, technologies or services within capitalist markets. Agroecology, with its emphasis on principles such as ecological processes, low external inputs, the agency of food producers and consumers, and autonomy from elite and corporate power, sharply contrasts with the incentives, policies, programmes, rules and norms of the dominant regime (Smith and Raven 2012).

In our framework, agroecology as a ‘niche’ does not only refer to single projects, clusters or localized experiments; it also embraces proto-regimes or counter-regimes that emerge from such local projects and inform them in turn. Within the MLP, such local experiments take place in ‘protective space’ on farms and in communities, shielded from a potentially hostile dominant regime through, for example, exemptions from regulations. Political agroecology is explicitly constructed to contest, deconstruct, transform and replace the dominant regime and is often born of radical demands for food sovereignty (Nyeleni Movement for Food Sovereignty 2007). Using this interpretation of niche level helps us to move analytically from seeing isolated ‘innovations’ in local niches as disconnected phenomena to seeing how—even when geographically separated—the experiments and experiences of niches can be a part of dynamics and movements working across space and time in processes of transformation.

As the concerns and positionality of political agroecology can be seen reflected in the demands of food sovereignty, let us look at those demands, as laid out in the 2007 Nyeleni Declaration:

1. A focus on food for people, with rights to sufficient, healthy and culturally appropriate food at the centre of food policies rejecting the treatment of food as just another commodity produced for the purpose of profit and the concomitant immorality of access to food depending on economic resources
2. Valuing food providers, particularly with regard to securing rights and respect for those who grow, harvest and process most of the world’s food: farmers and workers within small-scale, family, traditional and indigenous food systems
3. The localization of food systems, *inter alia*, in contrast to the currents of capital favouring large corporations
4. Local control of food providers and consumers over territory, land, grazing, water, seeds, livestock and fisheries based on the rights of local food producers and inhabitants in territory—food sovereignty rejects the privatization of such resources, for example through intellectual property rights regimes or commercial contracts
5. Broad-based skill-building that supports indigenous knowledge in local communities, in part through the management, conservation and development of local food production, harvesting and distribution systems and appropriate research supporting these activities
6. Working with nature by respecting and supporting the integrity and contributions of ecosystems and communities’ ecological knowledge, particularly the use of diversified agricultural methods reliant on few external inputs
  In Canada, a seventh principle was added by the Indigenous Circle of the People’s Food Policy:
7. Food is sacred and not to be squandered.

This final demand counters the commodification of food and claims that the spiritual and cultural dimensions of food are fundamentally important: food is central to who we are as people. It reflects an indigenous cosmovision based on respect towards nature.

Political agroecology, as the conceptual basis for putting agroecology and food sovereignty into practice, is not the only possible formulation of such a niche. Others have claimed organic, sustainable or climate-smart agriculture, alternative food networks and other systems as alternatives. While related to agroecology, these systems are not always clearly allied to political transformation; they often leave existing power dynamics in place and in many ways reinforce the dominant regime and the position of actors in it. Some approaches, such as organic agriculture, had a radical and transformative agenda that, over time, has been twisted to conform to the dominant regime in many respects (even if a radical movement does continue to exist alongside the more mainstream dimensions of organic agriculture and markets). As a radical alternative, agroecology is thus also competing with niches that are much more aligned to and supported by the dominant regime.

While agroecology and food sovereignty are not immune from being co-opted or deployed with a view of superficial reforms (as will be discussed later in the book), they are currently the most significant, well-developed and coherent formulations for advancing a counter-regime. Within the wider context of the world historical analysis provided by food regime theory, it is in this active formulation of political agroecology that social movements are pushing for emancipatory and transformative change—and that the social agency of affected peoples is realized from the bottom up.

### Our Approach: Advancing an Agency-Centric Approach to the Governance of Agroecology

In the MLP, transition is driven by interactions between the three levels, especially—as we have mentioned—niche and regime. While niches may influence the regime, the regime may act or react in ways that affect the niche’s growth. Generally speaking, as we have shown, regimes are configured to maintain the status quo and therefore may marginalize or co-opt emerging alternatives while actors within the regime work politically to maintain this position. This does not mean that socio-technological configurations such as regulations, policies and laws are fixed or that dominant regime actors want them to remain unchanged. On the contrary: changing, rearranging and tweaking rules, norms, technologies or laws often reinforce their power and position.

As such, issues of power and governance are critical but often underappreciated in the understanding of sustainability transitions through the MLP. Governance is the result of numerous interactions among actors in the government, private sector and civil society who, directly or indirectly, shape its content, interpretation and implementation. Omar Felipe Giraldo and Peter Rosset (2018) use the term “territory in dispute” for this battle in governance between institutions in the regime and social movements advancing agroecology as political struggle. This struggle plays out in economies, the environment and other material dimensions but also in the realm of ideas. Both are central to our analysis.

In examining how power and governance might be shifted to allow agroecology to fulfil its emancipatory potential, some questions can be helpful. Which actors are involved? Where does ‘governance-making’ actually take place? Who has final control over decision-making processes? Whose perspectives, knowledge, values and aspirations are embedded in governance and whose are excluded? Through which avenues can governance be improved? Whose interests are served, and is someone held accountable? Asking these questions helps to shift attention from an analysis of governance per se to the analysis of the governance *process*. This is important because, given the often-contested pathways and goals of agroecological transformation, governance must be equitable.

Wider political, economic, social, cultural and ethical contexts—as well as norms and rules of power—shape any governance system for agroecological transitions. The architecture of such systems covers a spectrum, from highly centralized, uniform, top-down and coercive decision-making to decentralized, horizontally distributed, participatory forms that are socially inclusive and tailored to local contexts (Pimbert 2018). Some prevailing governmental systems are highly prejudicial, excluding, disempowering and oppressing groups including women, people of colour, ethnic minorities, indigenous peoples, LGBTQ+ groups and youths. Thus, when we refer to confronting an unjust and unsustainable dominant regime, we are also referring to its systemic racism and patriarchal, heteronormative stance (see especially Chap. 10.1007/978-3-030-61315-0_8 on equity).

It is clear that corporate regimes lock in many unjust aspects of governance of food and agriculture and that these ultimately influence all emerging alternatives or attempts for autonomy. In our view, this is inescapable. Any notion of building completely autonomous alternatives is naïve, at base. Moreover, engaging in transformative action at the niche-regime interface involves a fraught engagement with the power of the regime, which often operates in subtle, hidden and systemic ways (Laforge et al. 2016). Collective efforts to shift power thus demands care and awareness of any implications of various actions and interventions.

While the tensions, contradictions and risks are very real in these governance interventions, so too are the possibilities for transformation. Even agroecology niches that flourish on their own terms may not contribute to transformation if the regime and its governance are not challenged. The parts on the domains of transformation (Part II) and on the six ‘effects’ of governance interventions (Part III, chapter 10) offer insights into how this can be accomplished.

Finally, a word about scale in relation to governance. Complex forms of governance occur across multiple interacting scales, and the enactment of power at one scale is influenced by the relationship between actors and institutions at others—household, local, territorial, national and international. For instance, international trade agreements, such as the North American Free Trade Agreement (NAFTA), will shape possibilities for developing alternative food networks in specific territories and will also have implications for individual households. While a deep dive into these dynamics is beyond the scope of this book, we will share insights on them in examples.

In part III we present the territorial scale as decisive in food systems transformation. Defined by a range of spatial, environmental, political and cultural factors, the territory is a key meeting place for actors, sectors, ideas and practices and for the interaction of food producers’ strategies with state policies. But any efforts to enable a territorial approach also demands work at other levels of governance—in part, to transform relationships and structures at those scales (e.g. global) where political power has congealed.

## Introducing Domains of Transformation

To better understand and construct an agency-centred approach to agroecology transformations, we have developed the notion of the *domains of transformation*. These domains represent discrete spheres of activity within which agroecology (a ‘niche’ in MLP terms) and the dominant regime come into conflict. Parsing out these niche-regime interactions into domains helps to break down the transformation pathway into key areas of intervention. However, it is vitally important to understand that these domains are interrelated and should not be viewed in isolation. An intervention in one domain often has an effect in another domain, as we will illustrate below. Thus, the overlap and alignment of domains is critical in accelerating agroecological transformation, which calls for a holistic and cross-domain analysis and transformation strategy.

**Fig. 3.2:**
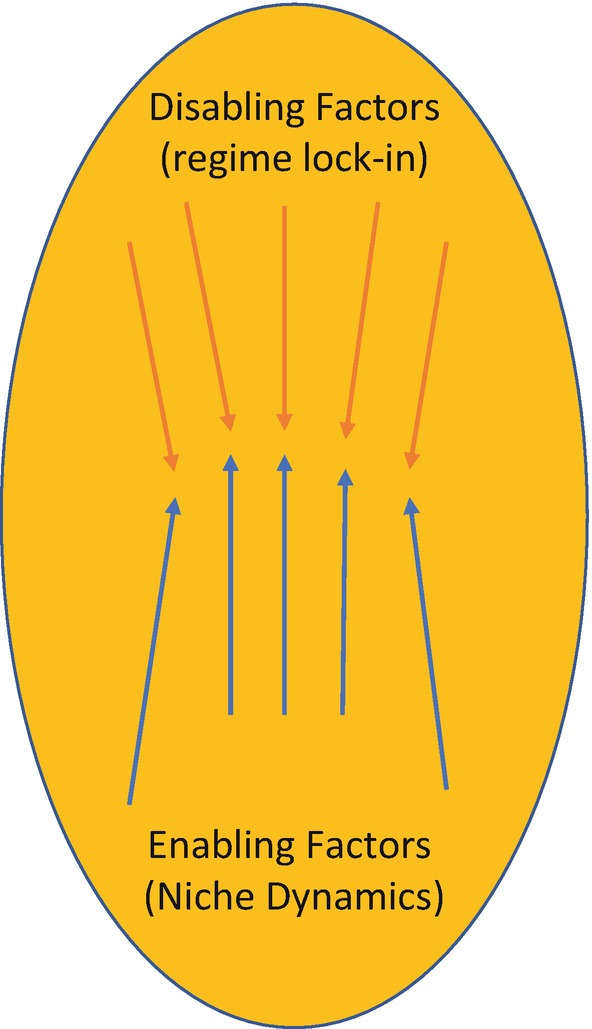
Within each domain, there are factors, dynamics, structures and processes that constrain agroecology (orange examines these dynamics within six arrows), and those that enable it (blue arrows). Our analysis shows interdependent domains of transformation (see Fig. 3.4)

**Fig. 3.3:**
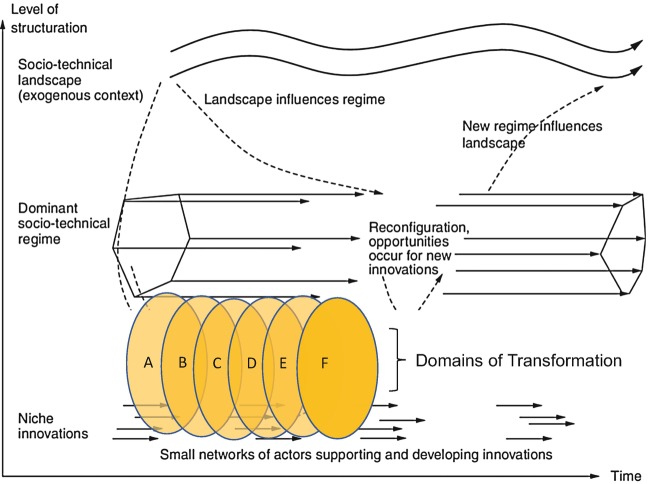
Domains of transformation are depicted here as definable interfaces between niche and regime superimposed onto a simplified version of Frank Geels and Johan Schot’s (2007) multi-level perspective figure

We derived these domains through a process of collectively analysing the growing literature and case studies on agroecology transitions (see Anderson et al. 2019 for a more detailed methodology). Through an iterative process of collective reading, group discussion and diagramming our emerging analysis across these studies, we developed our framework of six domains of transformation. We started by identifying the enabling factors and disabling factors that we found in each study. As we viewed the emerging patterns across the studies it became clear that there were six main domains within which the majority of the enabling and disabling factors could be attributed. It also became clear that these domains were situated at the intersection of and conflict between the niche—where proponents of agroecology are strategically working to enable agroecology—and the regime—where inertia of the dominant system and resistance by dominant actors disable agroecology (Figs. 3.2 and 3.3).

**Fig. 3.4:**
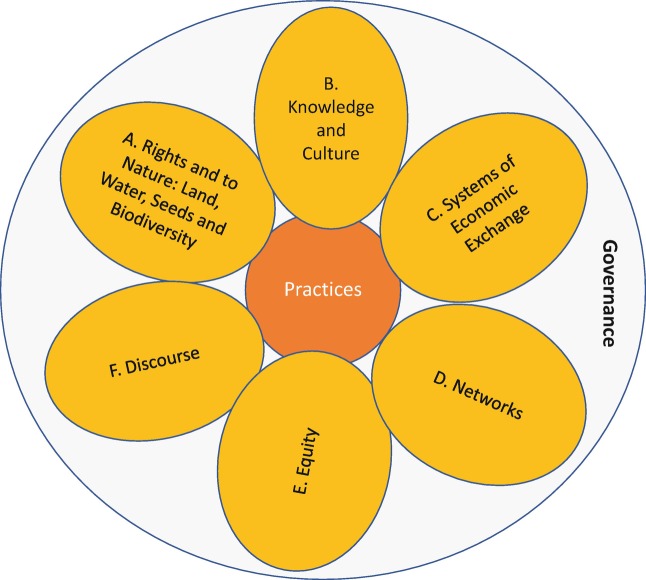
These six domains of transformation, within which agroecology comes into conflict with the dominant corporate food regime, are critical sites of intervention in pursuit of agroecology transformations. The extent and depth of agroecological practices on farms and in territories are shaped by processes of governance, power and control as they manifest in and across these domains

The six domains (Fig. 3.4) that are critical in agroecological transformations are: rights and access to nature, knowledge and culture, systems of economic exchange, networks, equity and discourse. In Part II, we analyse the enabling and disabling dynamics within each of these domains. We will then go on to discuss in Part III how, in these domains, transformations in governance and power relationships can gain strength, gradually enabling agroecology—a new, more just food regime—to take root.
